# Structural basis of σ^54^ displacement and promoter escape in bacterial transcription

**DOI:** 10.1073/pnas.2309670120

**Published:** 2024-01-03

**Authors:** Forson Gao, Fuzhou Ye, Bowen Zhang, Nora Cronin, Martin Buck, Xiaodong Zhang

**Affiliations:** ^a^Section of Structural and Synthetic Biology, Department of Infectious Disease, Imperial College London, London SW7 2AZ, United Kingdom; ^b^London Consortium for High Resolution cryoEM, the Francis Crick Institute, London NW1 1AT, United Kingdom; ^c^Department of Life Sciences, Imperial College London, London SW7 2AZ, United Kingdom; ^d^DNA processing machines laboratory, the Francis Crick Institute, London NW1 1AT, United Kingdom

**Keywords:** RNA polymerase, σ factor, transcription initiation, σ^54^, cryo-electron microscopy

## Abstract

Gene transcription is a fundamental cellular process carried out by RNA polymerase (RNAP). Transcription initiation is highly regulated, and in bacteria, transcription initiation is mediated by sigma (σ) factors. σ recruits RNAP to the promoter DNA region, located upstream of the transcription start site (TSS) and facilitates open complex formation, where double-stranded DNA is opened up into a transcription bubble and template strand DNA is positioned inside RNAP for initial RNA synthesis. During initial transcription, RNAP remains bound to σ and upstream DNA, presumably with an enlarging transcription bubble. The release of RNAP from upstream DNA is required for promoter escape and processive transcription elongation. Bacteria sigma factors can be broadly separated into two classes with the majority belonging to the σ^70^ class, represented by the σ^70^ that regulates housekeeping genes. σ^54^ forms a class on its own and regulates stress response genes. Extensive studies on σ^70^ have revealed the molecular mechanisms of the σ^70^ dependent process while how σ^54^ transitions from initial transcription to elongation is currently unknown. Here, we present a series of cryo-electron microscopy structures of the RNAP-σ^54^ initial transcribing complexes with progressively longer RNA, which reveal structural changes that lead to promoter escape. Our data show that initially, the transcription bubble enlarges, DNA strands scrunch, reducing the interactions between σ^54^ and DNA strands in the transcription bubble. RNA extension and further DNA scrunching help to release RNAP from σ^54^ and upstream DNA, enabling the transition to elongation.

## Significance

Gene transcription is carried out by RNAP which is recruited to transcription start sites (TSS) via specific factors. In bacteria, sigma factors recruit RNAP by binding to gene promoter sites upstream of TSS. Once a short stretch of nucleotides is synthesized, RNAP needs to escape from the promoter site so that it can translocate downstream to continue RNA synthesis. There are two major sigma factor classes, σ^70^ and σ^54^, which are structurally and functionally distinct. Thus far, we have limited information on how promoter escape occurs in the major variant σ^54^ system. Here, we present several cryo-electron microscopy structures of the RNAP-σ^54^ initial transcribing complexes and propose a molecular mechanism for how RNAP escapes from promoters in this system.

**Fig. 1.**
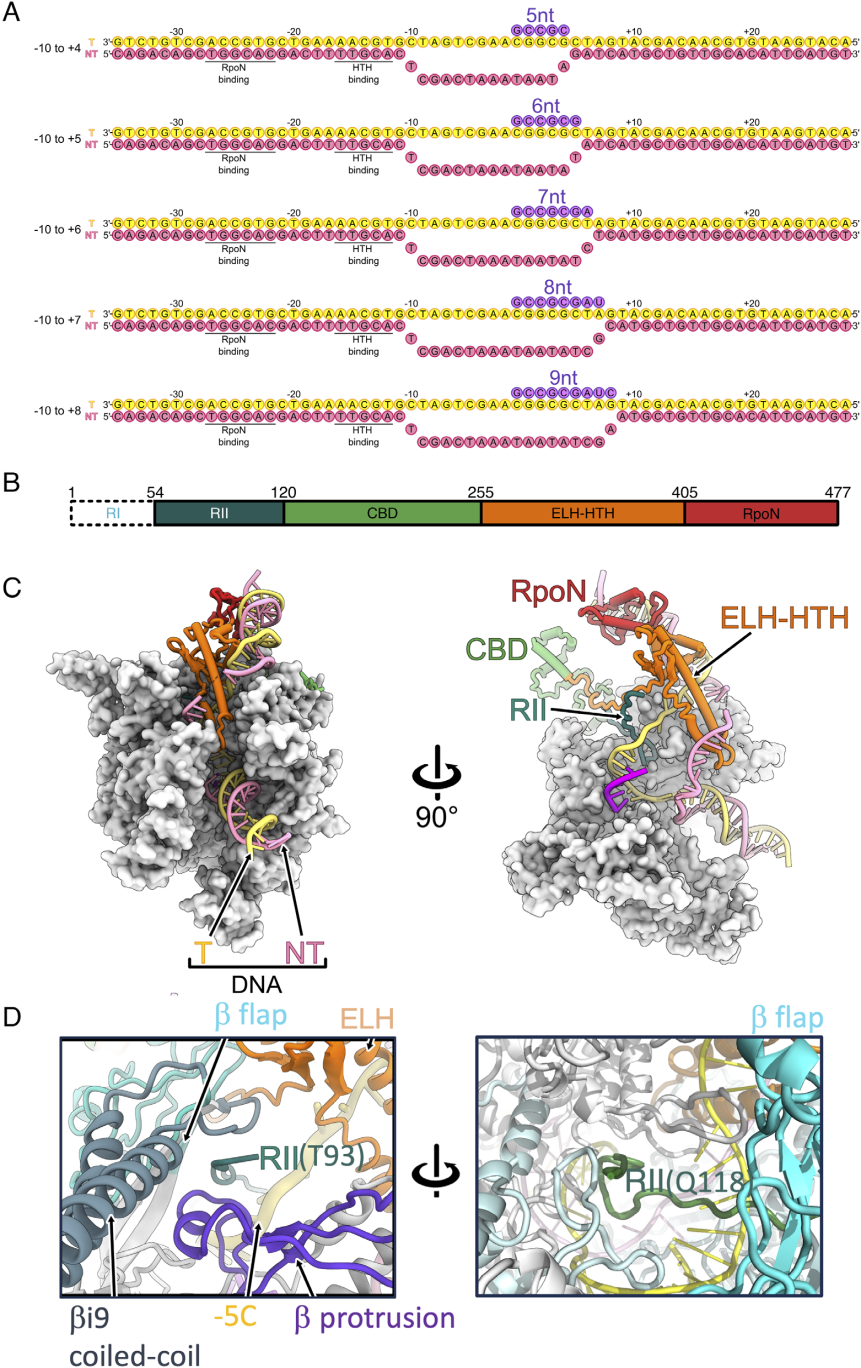
Overall structures and nucleotides used in this study. (*A*) Design of the DNA–RNA scaffolds used in this study, showing complete DNA/RNA nucleotide sequences. (*B*) Overview of the σ^54^ domain structure. RI is shown in white, as it is unresolved. (*C*) *Left*: overview of 5 nt initial transcribing complex (RPitc-5nt) structure, viewed from the downstream. RNAP is shown in white. σ^54^ domains colored as in (*B*), template strand: yellow, nontemplate strand: pink, RNA: magenta. *Right*: cross-section of RPitc-5nt, with RII highlighted in dark green, α and β subunits are hidden for clarity. (*D*) RII enters and exits the RNAP core. *Left*: RII enters RNAP via a site surrounded by the β protrusion, β flap, β i9 coiled-coil, and ELH-HTH subdomains. *Right*: RII exits RNAP via the RNA exit channel. The two sites are separated by β flap.

The multisubunit RNA Polymerases (RNAPs) are conserved from bacteria to human, with the active site located inside the RNAP cleft formed by the two highly conserved large subunits (β and β′ in bacteria) (1) (Fig. 1). Transcription initiation in bacteria involves the recruitment of RNAP by sigma factors (σ) to promoter regions located upstream from the transcription start site (TSS, position +1) (2). Bacteria usually have several σ factors with the majority belonging to the σ^70^ family, represented by the housekeeping σ factor (3). In approximately 60% of bacteria, a major variant σ factor, σ^54^, forms a class of its own, lacking structural and sequence similarity to σ^70^. In σ^54^-mediated transcription initiation, σ^54^ recruits RNAP to genes responsible for regulating a variety of stress responses, including nutrient depletion, membrane stress, antibiotic exposure, and biofilm formation (4). Once recruited, the RNAP-σ holoenzyme forms a closed complex at promoter DNA, which remains duplexed and outside the RNAP cleft. The closed complex is subsequently converted to an open complex, where the duplex DNA strands are separated into a transcription bubble and the template strand is delivered into the RNAP cleft with the TSS positioned at the active site, ready for transcription (5). Biochemical, biophysical, and structural studies have shown that the transcription bubble consists of ~13 nt single-stranded DNA (between −11 and +2 relative to the TSS) for both σ^70^- and σ^54^-dependent transcription, despite the different mechanisms for maintaining the transcription bubble [see reviews (4, 6)]. During initial RNA synthesis, the upstream DNA is shown to remain bound by RNAP-σ^70^ while DNA downstream of TSS is brought to the RNAP active site, thus resulting in an enlarged transcription bubble inside the RNAP cleft (7–9). Biochemical and structural studies show that RNAP-σ^54^ also remains bound to upstream DNA, and the upstream edge of the transcription bubble, DNA strands at −10, remains separated during initial transcription (10, 11), suggesting that similar to RNAP-σ^70^, downstream DNA is also brought into the RNAP cleft for initial transcription in RNAP-σ^54^.

Promoter escape is the final step in transcription initiation, when RNA reaches sufficient length, RNAP is released from the promoter region and translocates downstream. During elongation, the transcription bubble collapses to 10 nt and is maintained by interactions with a series of conserved regions in the RNAP core (*SI Appendix*, Fig. S1, discussed in more detail in ref. 12). During elongation, the DNA–RNA hybrid is cordoned between the highly conserved β′ bridge helix and the β′ lid. The RNA exit channel is made up of the β′ zipper, β flap, and β switch three loops (*SI Appendix*, Fig. S1).

Although σ^70^ and σ^54^ are structurally and mechanistically distinct, they contain subdomains serving analogous functions (Fig. 1*B* for σ^54^) (13). Both σ factors contain flexible linkers that descend into the active cleft of the RNAP and occupy the RNA exit channel (for σ^54^, see Fig. 1*C*). In the RNAP-σ^70^ holoenzyme and closed promoter complex, region 1.1 occupies the site where downstream DNA resides in the elongation complex, whilst region 3.2, also known as the σ finger, occupies the RNA exit channel (14, 15). In the RNAP-σ^54^ holoenzyme, region II (RII) occupies both the downstream DNA binding site and the RNA exit channel, with the core binding domain (CBD) occupying the site where RNA exits (13, 16), interacting with the RNAP α-CTD, β flap, β′ zinc finger (Fig. 1 *C* and *D* and *SI Appendix*, Fig. S1) (13).

Previous studies have revealed that in the σ^70^-depenedent system, transition into the elongation complex involves σ domain relocation (17). Structural, biochemical, and biophysical studies show that during initial transcription, RNA extension causes the 5′ end of the RNA to push against the σ finger (18, 19). Furthermore, it has been proposed that energy stored in DNA during DNA scrunching would be released, helping with promoter escape (7, 8, 20). It is unclear if and when σ^70^ is released from RNAP during elongation, and whether the release step is DNA sequence-dependent.

Given the structural differences between σ^54^ and σ^70^, it is thus unclear how σ^54^ relocation occurs during initial transcription and whether σ^54^ needs to dissociate from RNAP to proceed to elongation. In this study, we used single-particle cryo-electron microscopy (cryoEM) to determine the structures of initial transcribing complexes of increasing RNA lengths from 5 nt to 9 nt, which allow us to propose a mechanism of RNAP promoter escape in the σ^54^ system.

## Results

**Fig. 2.**
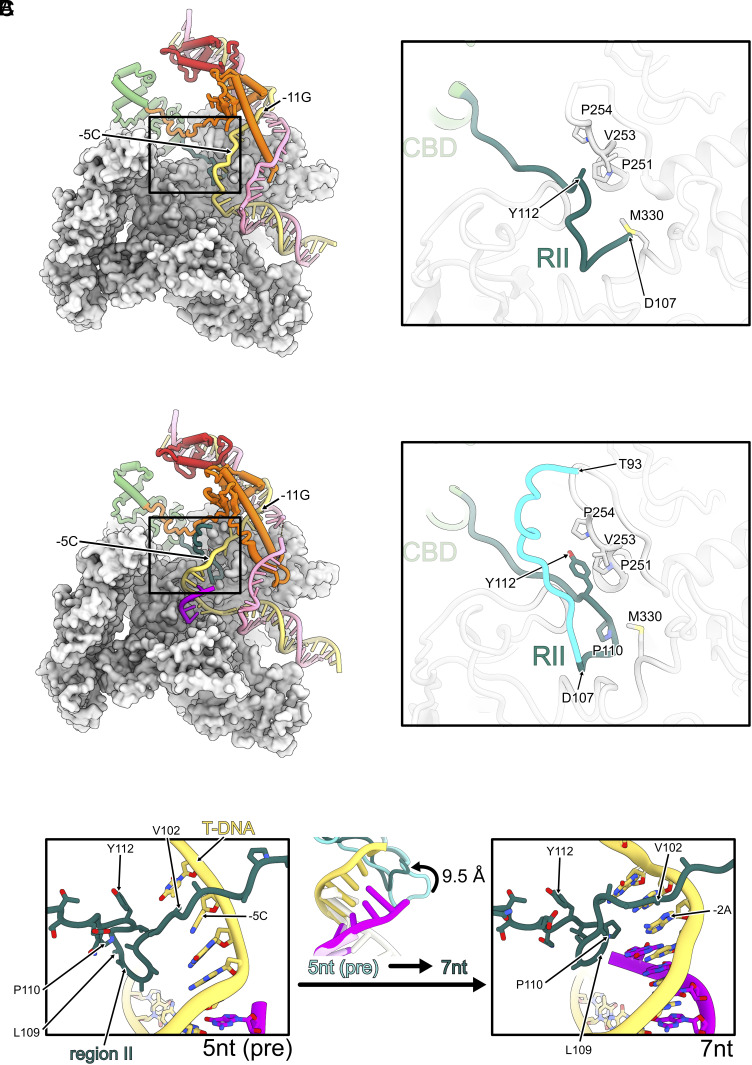
Region II-finger and transcription bubbles in RPitc. (*A*) Cut open view of the open complex structure (RPo), *Right*: zoomed in view showing RII tip is only partially resolved (*B*) Same view as (*A*) in the 5 nt initial transcribing complex (RPitc-5nt). RII tip is resolved and kinks sharply at the site close to the template strand DNA. Residues resolved in RPitc-5nt that is absent in RPo are colored in cyan. Key residues are labeled. (*C*) RII tips and interactions with DNA template-strand in RPitc-5nt (*Left*) and RPitc-7nt (*Right*) complexes. Key residues are labeled. The RII tip folds back 9.5 Å in RPitc-7nt compared with RPitc-7nt (*Middle*).

To capture structures of initial transcribing complexes that lead to promoter escape, we designed DNA–RNA scaffolds based on the well-characterized *nifH* promoters. The scaffolds contained increasing sizes of transcription bubble and RNA lengths to mimic the growing mRNA and the corresponding enlarged transcription bubble as observed in σ^70^ system and proposed for σ^54^ system since interactions with upstream DNA remain unchanged (Fig. 1*A*) (10, 16). Using cryoEM and single-particle analysis (Figs. 1 and 2, Tables1 and 2, and *SI Appendix*, Figs. S2–S6), we determined structures of initial transcription complexes (RPitc) containing RNA lengths of 5, 6, 7, 8, and 9 nt.

**Table 1.** cryo-electron microscopy data collection parameters

Sample	Pixel size (Å/pixel)	Total dose (e^−^/Å^2^)	Defocus range (μm)	Total movies
**RPitc 5nt**	1.1	30	−3.0 to −1.0	12,309
**RPitc 6nt**	1.06	40	−2.7 to −1.0	9,188
**RPitc 7nt**	1.06	30	−3.0 to −1.0	4,185
**RPitc 8nt**	1.072	40	−2.7 to −1.0	11,198
**RPitc 9nt**	1.06	30	−3.0 to −1.0	6,121

**Table 2.** Model validation statistics for the initial transcription complexes

Sample	RPitc 5nt pre-translocated	RPitc 5nt post-translocated	RPitc 6nt	RPitc 7nt	RPitc 8nt	RPitc 9nt
**Refinement**						
Resolution (Å)	2.8	3.4	3.4	3.5	3.9	3.8
Initial number of particles	1,624,791	1,624,791	705,223	654,448	998,879	491,140
Final number of particles	571,698	27,465	73,619	46,380	28,569	13,704
**Model composition**						
Nonhydrogen atoms	29,191	28,821	28,681	28,965	28,878	28,605
Protein residues	3,668	3,668	3,638	3,635	3,617	3,617
Nucleic acid residues	102	100	101	104	105	103
Ligands	2 Zn^2+^	2 Zn^2+^	2 Zn^2+^	2 Zn^2+^	2 Zn^2+^	2 Zn^2+^
	1 Mg^2+^	1 Mg^2+^	1 Mg^2+^	1 Mg^2+^	1 Mg^2+^	1 Mg^2+^
**B factors (Å^2^)**						
Protein	63.87	40.32	63.05	50.02	42	37.68
Nucleic acid	23.49	95.31	101.33	101.62	156.55	180.21
Ligands	69.42	27.99	58.31	54.39	33.57	46.53
**RMS deviations**						
Bond lengths (Å)	0.004	0.003	0.005	0.005	0.005	0.003
Bond angles (°)	0.679	0.604	0.71	0.765	0.681	0.643
**Validation**						
MolProbity score	1.94	1.85	1.99	1.96	1.95	1.95
Clashscore	10.87	8.71	10.84	11.06	10.12	11.03
Poor rotamers (%)	0.59	0.27	0.42	0.37	0.37	0.34
**Ramachandran plot**						
Favored (%)	94.29	94.37	93.3	94.13	93.59	94.28
Disallowed (%)	0	0.03	0.03	0.03	0.03	0.08

### Structures of RPitc with 5 nt mRNA Identify the RII-Finger.

Using a combination of focused classification and electron density subtraction, we obtained several distinct structural states within the same dataset, including pre- and post-translocated RNA states, as well as σ-free states (*SI Appendix*, Fig. S4). We focused on the pre- and post-translocated states which were resolved to 2.8 and 3.4 Å resolution respectively based on gold standard Fourier Shell Correlation curves (*SI Appendix*, Figs. S3 and S4) (21).

We could resolve almost the entire RNAP (including one α-CTD), DNA between −29 and +20, 5 nt RNA as well as σ^54^ CBD, ELH-HTH, and RpoN domains (Fig. 1 and *SI Appendix*, Figs. S2–S6). Similar to our previously published open complex (RPo) and initial transcribing complex (4 nt, RPitc-4nt) structures (16), we could not resolve RI and the N-terminal part of RII (~ first 110 residues of σ^54^, Fig. 1*B*), due to their flexible nature in these complexes. The C-terminal part of RII inserts into the RNAP cleft and comes in close proximity to the template strand DNA and RNA–DNA hybrid (Fig. 1*C*). RII links RI and CBD, the C-terminal portion of RII occupies the RNA exit channel, leading to CBD that resides at the RNA exit (Fig. 1*C* and *SI Appendix*, Fig. S1). The remaining visible part of RII emerges from a channel formed by β flap, βi9 coiled-coil, and β-protrusion, toward the protein surface, presumably linking to RI via the flexible N-terminal part of RII (Fig. 1 *D*, *Left*). Indeed, RII enters and exists RNAP via two separate regions in RNAP, separated by β flap and βi9 coiled-coil (Fig. 1*D*).

Compared to the RPo structure, the RNAP and σ^54^ domains remain largely unchanged (Fig. 2 *A* and *B*) (16). In RPo, we could only resolve RII residues from D107 onwards (Fig. 2 *A*, *Inset*). In the RPitc-5nt structures, we could resolve extra residues in RII, including 93–106 (Fig. 2 *B*, *Inset*). The RII residues C-terminus to Y112 are in similar conformations. In RPitc-5nt, residues 104–112 form the tip of a β-hairpin (Fig. 2 *B*, *Inset*), and residues 93–103 emerge from the β subunit side of the RNAP instead (Fig. 1 *D*, *Left*). Interestingly, this is different from the RII trace in the RNAP-σ^54^ holoenzyme and closed complex, where this part of RII occupies the template strand position, just above the bridge helix (*SI Appendix*, Fig. S7*A*) (13). Presumably upon open complex formation and during initial transcription, RII relocates to make space for the transcription bubble. In RPitc-5nt, RII contacts the template strand at the −5 position (Fig. 2*C*), and threads around the template DNA–RNA hybrid and forms a sharp kink at P110 (Fig. 2 *B*, *Inset*). Indeed, the bases of the template strand interact with RII (Fig. 2 *C*, *Left*). We now refer to this β-hairpin (residues 104–112) as the RII-finger due to its functional similarity with the σ^70^ finger, also known as R3.2 in σ^70^. The RII-finger is stabilized by hydrophobic interactions between P110 and Y112 of RII, and the conserved P251, V253, and P254 on the β′ lid and M330 on β′ switch 2 region (Fig. 2 *B*, *Inset*).

### RNA Extension Causes Folding Back of the RII-Finger.

**Fig. 3.**
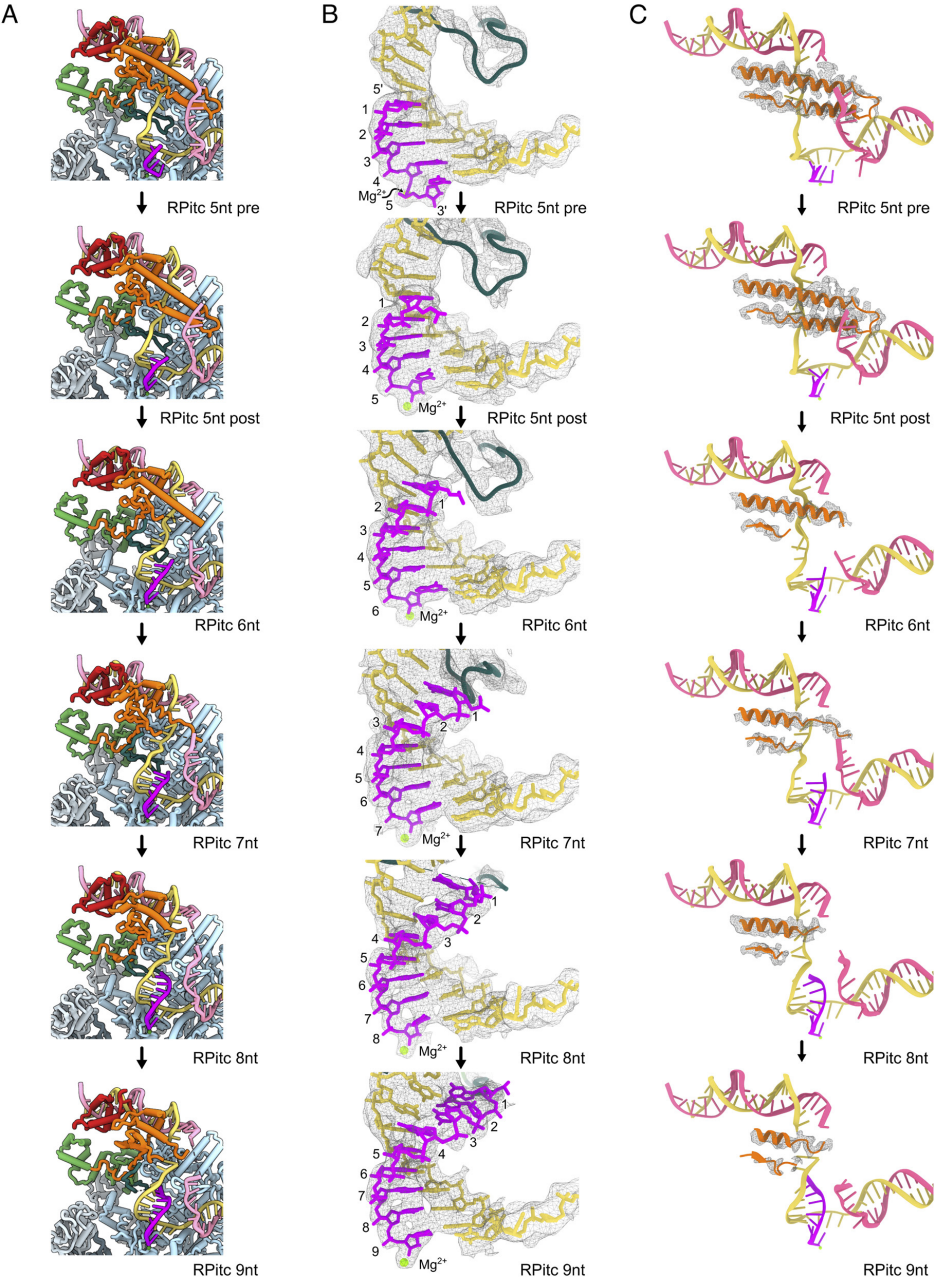
Conformational changes during initial transcription. (*A*) Overall structures of the initial transcribing complexes from 5 nt to 9 nt RNA. β subunit was omitted in these figures for clarity. (*B*) Electron density for RNA, template strand DNA, and RII-finger at different transcript lengths. At 7 nt, RII-finger folds back to accommodate the enlarged DNA–RNA hybrid. (*C*) Electron density for ELH at different transcription lengths.

Data collected on each of the initial transcribing complexes (Fig. 1*A*) were processed using similar strategies as for the RPitc-5nt dataset. In all the datasets, in addition to RNA-bound, post-translocated, σ^54^-bound states, we found a subset of σ^54^-free states (Fig. 3*A* and *SI Appendix*, Figs. S4 and S5). The σ^54^-free states are partly due to complex dissociation during cryo-electron microscopy sample preparation, which is shown to disrupt/destabilize macromolecular complexes (22). Interestingly, the proportion of σ^54^-free complexes increases with increasing RNA lengths (*SI Appendix*, Figs. S4 and S5), suggesting that the σ^54^-RNAP complexes become increasingly unstable.

The RII-finger conformation is almost identical between 6 and 5 nt and indeed RNA remains short of reaching RII-finger (Fig. 3 *A* and *B*). However, at 7 nt RNA, the RII-finger adapts a different conformation, to accommodate for the growing DNA–RNA hybrid and prevent steric clashes (Fig. 3*B*). The tip of the RII-finger is coiled backward by 9.5 Å, as measured from the Cα atoms of the tip of the RII-finger (Fig. 2 *C*, *Middle*). In RPitc-7nt, the folded back RII tip is stabilized by interactions between Y112 of RII and V253 of β′, P110 with the DNA–RNA bases (Fig. 2 *C*, *Right*). In 8 and 9 nt complexes, the density for the RII-finger is partially lost (Fig. 3), indicating this region becoming dynamic in nature and the interactions observed in 7 nt are lost. In addition, P110 and Y112, observed in 5 to 7nt complexes, were also not resolved, indicating that these interactions are no longer stably maintained. These results suggest that the folded-back conformation of the RII-finger observed in 7 nt complex is stabilized by specific hydrophobic interactions and therefore likely represents a kinetic barrier during initial transcription. This observation supports earlier biochemical data showing that when transcription substrates were limiting, RNAP-σ^54^ synthesized short transcripts up to 7 nt (10).

Taken together, data presented here suggest that RNA extension to 7 nt causes the folding back of the RII-finger, which is stabilized by specific interactions between RNA, DNA, and RII-finger. Further RNA extension releases RII-finger by breaking the hydrophobic interactions between the β′ lid and RII residues P110 and Y112.

### RNA Extension Drives DNA Scrunching, Altering Interactions between Templates Strand DNA and RII-Finger.

In RPo, the transcription bubble is separated by ELH (Fig. 2*A*) (16). During initial transcription, as in RPitc-5nt, the template strand expands into the back of the cleft (*SI Appendix*, Fig. S1*D*). The template strand now interacts with the σ^54^ RII-finger (Fig. 2*C* and *SI Appendix*, Fig. S1). In RPitc-6nt and RPitc-7nt, the template strand (at −5 and −6 positions) is scrunched around the RII-finger and σ^54^ ELH (*SI Appendix*, Figs. S1*D* and S3*C*), while the nontemplate strand scrunches around the ELH, resulting in poorly defined density (Fig. 3 *B* and *C*). At RPitc-8nt and -9nt, both template and nontemplate strands scrunch around the ELH, losing interactions between the DNA strands and ELH, resulting in reduced density for both DNA and the ELH (Fig. 3*C*). Our results thus suggest that DNA scrunching is a major mechanism during initial transcription up to 9 nt with progressively more scrunches occurring, initially close to the active site and then move upstream toward the σ^54^ ELH.

DNA upstream of −11 remains duplexed and unchanged during initial transcription. In vivo footprinting data show that DNA strands at −10 remain separated during initial transcription (10, 11), supporting our observation here that transcription bubble remains unchanged at the upstream edge. Instead, initial transcription causes DNA in the transcription bubble to scrunch in order to accommodate the enlarged transcription bubble.

### RNA Extension Increases the Dynamics of Transcription Bubble and σ^54^ ELH.

In addition to the RII-finger folding back and DNA scrunching, with the increased transcription bubble and DNA–RNA hybrid size, density for both ELH and the transcription bubble surrounding it become less well defined (Fig. 3*C* and *SI Appendix*, Fig. S6). The ELH density becomes significantly poorer at 6 nt compared to that of 5 nt, indicating that there is an increased flexibility of ELH from 6nt (Fig. 3*C* and *SI Appendix*, Fig. S6). ELH density continues to deteriote with increasing RNA lengths, and this coincides with the lack of continuous density for the nontemplate strand around here (*SI Appendix*, Fig. S6). Indeed, during initial transcription, apart from minor scrunching at the −1 position (nucleotide immediate upstream from the synthesizing site), the nontemplate strand mainly scrunches around ELH (Fig. 3).

ELH separates the DNA strands from −11 to −7 in the transcription bubble (Fig. 2*A*). Apart from the interactions with DNA, the ELH has very few interactions with the rest of RNAP in the cleft, suggesting that the ELH and the transcription bubble stabilize each other via their direct interactions. ELH flexibility thus can in part be a result of the enlarged transcription bubble, and the subsequent reduced interactions and constraints between the transcription bubble and ELH. The less constrained ELH will be able to slide and potentially retract out from the transcription bubble. Furthermore, we also observe the loss of resolution of the upstream DNA and upstream DNA binding domains on σ^54^ (RpoN, ELH-HTH) (*SI Appendix*, Fig. S6), suggesting that both the σ^54^ upstream DNA binding subdomains and upstream DNA are less stably engaged with RNAP.

## Discussion

### σ^54^-Dependent Promoter Escape Results from DNA Scrunching and RII Conformational Changes.

**Fig. 4.**
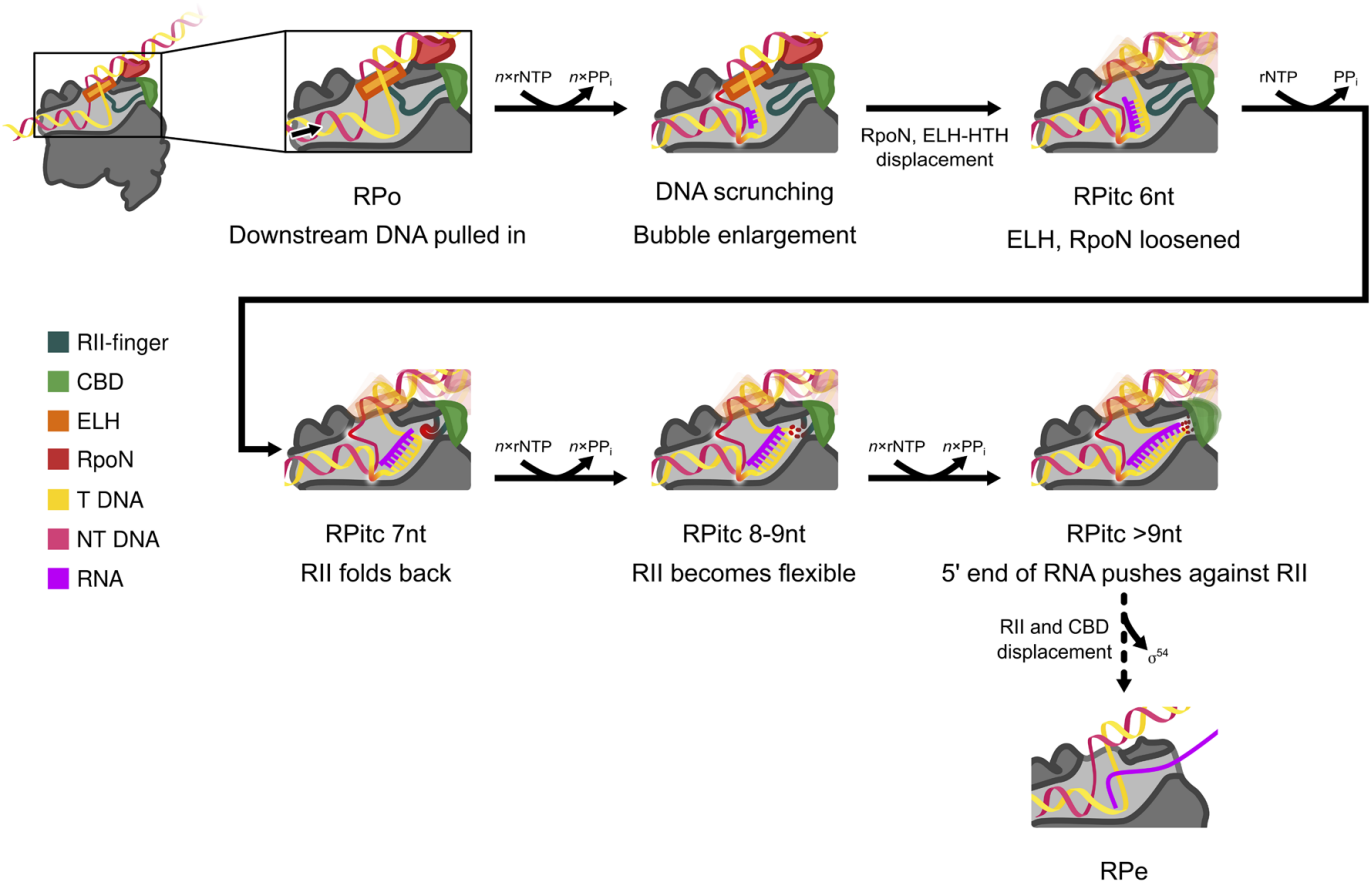
Proposed mechanism of promoter escape in RNAP-σ^54^. In RPo, the transcription bubble is separated and stabilized by ELH as well as conserved regions in RNAP. Initial transcription results in local DNA scrunching while the structure of RNAP-σ^54^ remains largely unchanged, with RII-finger inserted close to the template DNA strand and interact with template strand and RNAP. From 6 nt, DNA scrunching reduces interactions with ELH, enabling ELH to be more flexible. At 7 nt, RII-finger folds back and is stabilized by specific interactions with template strand DNA, RNA, and RNAP. From 8 nt, RII-finger is no longer stabilized, enabling growing RNA to extend in the RNA exit channel, eventually leading to the displacement of RII and CBD. ELH and DNA binding domains of σ^54^ become less associated with RNAP, eventually leading to RNAP escape from promoter sites.

The structures of RPitc complexes presented here suggest that during initial transcription, DNA upstream of the transcription bubble remains bound by RNAP-σ^54^, the downstream DNA is pushed into the catalytic site, and the transcription bubble enlarges inside the RNAP cleft (Fig. 4). Up to 6 nt RNA, the upstream DNA remains stably bound to the RNAP-σ holoenzyme. This is in agreement with models based on previous FRET and magnetic tweezers experiments and cryoEM studies of the σ^70^ system, demonstrating that RNAP remains bound to the upstream fork of the transcription bubble whilst downstream DNA is pulled in refs. 7 and 17. From 7 nt, ELH and upstream DNA become more flexible, indicating a global conformational change occurring within the transcription complex (Fig. 4).

The ELH interacts with and stabilizes the transcription bubble in the RPo structure (Fig. 2*A*, Fig. 3*C*) (16). With increasing RNA length (from 6 nt), the enlarged transcription bubble releases the interactions between the bubble and ELH, enabling it to eventually retrieve out of the transcription bubble. The movement of ELH could lead to reduced interactions between the three σ^54^ subdomains ELH-HTH-RpoN and the RNAP (Fig. 3*A*), as demonstrated by the reduced resolution of these three subdomains and increasing amount of RNAP complex without σ^54^ or DNA bound in the datasets, with increasing mRNA lengths (*SI Appendix*, Figs. S4–S6).

Given the extensive interactions between σ^54^ and upstream DNA (RpoN at −26 and ELH-HTH at −14, Fig. 3*A*), and the reduced interactions between σ^54^ and RNAP in the complex with longer RNA, it is possible that the stable attachment of σ^54^ to upstream DNA (−26 and −14) helps RNAP to be released from σ^54^ while translocating downstream, releasing the tension from the scrunched DNA, transitioning into the elongation complex. However, the conformational changes in σ^54^ upon RNAP dissociation, in particularly around RII and ELH, could also reduce σ^54^ attachment to promoter DNA, helping with σ^54^ release. Although in contrast with σ^70^, σ^54^ could bind to certain promoter DNAs alone (23, 24), whether σ^54^ remains bound to upstream DNA during elongation is currently unclear.

σ^54^ CBD interacts extensively with RNAP (Fig. 1*C*) (13, 16, 25) and does not interact with DNA directly. We did not observe significant movement of the regions that interact with CBD during initial transcription. This suggests that the initial transcription bubble enlargement and DNA scrunching are unlikely to significantly perturb the position of the CBD. This is indeed the case (Fig. 3*A*). However, elongating RNA, RII movement, movement of other σ^54^ domains or RNAP translocating downstream will ultimately lead to CBD dissociation from RNAP, and σ^54^ displacement from RNAP.

RI is the major inhibitory domain that prevents spontaneous open complex formation. RI is not resolved in these initial transcribing complex structures. From our previously determined closed and intermediate complex structures, we showed that RI contains a short helix that is stabilized by interactions with the -12/−11 promoter DNA region as well as hydrophobic and salt bridge interactions with the ELH. RI thus constrains ELH conformation and together they form an obstacle for DNA loading (25, 26). Recently, we had shown that RI N-terminal peptide is enclosed by the activator hexamer and the RI helix is proposed to be unfolded upon ATP hydrolysis of activators, releasing its constraint on ELH (26). In these initial transcribing complexes, RI is not resolved, presumably dissociated from ELH and is now flexible.

The results presented here also suggest a potential path for σ^54^ dissociation from RNAP. σ^54^ is deeply embedded in RNAP with RII entering and exiting from different parts of RNAP, implying σ^54^ dissociation is a complex process. RII, which links RI and RIII-CBD, inserts deeply into RNAP cleft. The N terminus of RII enters the catalytic site from the RNAP β side, in between the β protrusion, βi9 coiled-coil, β-flap, and σ^54^ HTH (Fig. 1 *D*, *Left*) while the C-terminus of RII exits via the RNA exit channel, between β and β′, towards the β′ side (Fig. 1 *D*, *Right*). The two exits are separated by β flap (Fig. 1*D*). During initial transcription, DNA scrunching results in reduced interactions between DNA and ELH, thus allowing ELH to be retrieved from the transcription bubble. RNA extension would cause CBD dissociation from RNAP. Given that CBD is held in position by interactions with β′ zinc-finger and β flap, CBD dissociation would cause the β flap to relocate, enabling the retrieval of RII from the RNAP cleft.

Interestingly, despite the important roles we have identified in promoter escape, RII is the least conserved regions in σ^54^ both in sequence identity and sequence lengths, with some species having a significantly shortened RII (for example in *Rhodobacter capsulatus*) (27). It is therefore possible that in these species, σ^54^ displacement occurs at longer RNA lengths and involves more DNA scrunching, similar to those observed in σ^H^ (ECF) factor (18). Furthermore, the specific interactions with template strand would suggest the dynamics and kinetics of σ^54^ displacement could be promoter sequence-dependent.

### Comparisons with σ^70^ Promoter Escape.

In σ^70^, as observed in X-ray crystallographic structures, the σ finger is seen to start to fold back in a stepwise fashion from 5 nt RNA (17). In σ^54^, we observe RII-finger folding back when RNA length reaches 7 nt to accommodate the increasing DNA–RNA hybrid. As RNA length increases to 9 nt, RII becomes more flexible. Our studies thus demonstrate that σ-finger refolding is likely a common mechanism for σ displacement, with R3.2 of σ^70^ being functionally analogous to σ^54^ RII-finger (18, 28). As the RNA extends, the RII-finger is pushed toward RNA exit, the CBD dissociates and the rest of RII would be pulled out of the active cleft (Fig. 4). On the other hand, σ^70^ core-binding domain R2 is not involved in DNA interactions or RNA extension, it is therefore consistent with σ^70^ sometimes remaining RNAP bound after promoter escape (29, 30).

Furthermore, scrunching of the DNA has been previously observed in single-molecule experiments with σ^70^ and has been proposed to also play a role in the build-up of stress within the walls of the RNAP catalytic site (31). We propose that scrunching of DNA in σ^54^ also plays a significant role, primarily in reducing the interactions with RII-finger and ELH, thus helping in releasing σ^54^.

Despite the functional and mechanistic similarities, there are differences between σ^54^ and σ^70^. R3.2 of σ^70^ enters deeper into the catalytic site compared to RII of σ^54^, consequently it starts to fold back when 5 nt RNA is synthesized while this only occurs with 7 nt RNA for RII-finger of σ^54^ (*SI Appendix*, Fig. S7*B*). Furthermore, DNA scrunching and RNA extension reduce interactions between DNA and RNAP and between σ^54^ and RNAP, while the interactions between σ^54^ and DNA at −26 and −14 remain unaffected. It is thus possible that promoter escape involves the translocating RNAP downstream while σ^54^ remains tethered to upstream −26 and −14 regions. This is in contrast with σ^70^, which sometimes remains RNAP bound after promoter escape (29, 30).

## Materials and Methods

### Protein Purification.

Protein purification was carried out as previously described, using a R336A mutant of σ^54^ that bypass the requirement of activators for open complex formation (16). RNAP-σ^54^_R336A_ was formed by incubating RNAP with σ^54^_R336A_ in a 1:4 molar ratio at 4 °C for 1 h, before gel filtration using a Superose 6 10/300 column (GE Healthcare) equilibrated with GF buffer (20 mM Tris-HCl pH 8, 150 mM NaCl, 5% v/v Glycerol, 2 mM TCEP).

### Design of DNA–RNA Scaffolds.

DNA and RNA were synthesized as single-stranded oligos by IDT and the oligos were annealed by mixing equimolar amounts of complementary strands in 20 mM Tris, pH 8.0 and heating to 95 °C for 2 min before cooling to 4 °C, by reducing 2 °C per minute. Oligos were used directly in cryoEM sample preparations.

### Sample Preparation.

First, 17 μM RNAP-σ^54^_R336A_ was incubated with 18.7 μM DNA–RNA scaffold in the presence of buffer EM1 (20 mM Tris-HCl, 150 mM NaCl, 10 mM MgCl_2_, 1 mM TCEP) for 1 h at 4 °C. Following incubation, samples were buffer exchanged into Buffer EM2 (20 mM Tris-HCl, 150 mM KCl, 5 mM MgCl_2_, 5 mM TCEP) using a 0.5 ml Zeba™ 7 K MWCO desalting column as per the manufacturer’s protocol. Then, 8 mM CHAPSO was then added immediately before cryoEM grid preparation.

### Grid Preparation.

First, 300 mesh holey gold C-flat R1.2/1.3 grids (ProtoChips) were plasma cleaned in air for 30 s (Harrick Plasma). Then, 4 μL of complex was deposited onto plasma-cleaned grids. The blotting parameters were as follows: wait time 30 s, blot time 2 s, and blot force −8. Grids were made using a Vitrobot™ Mark IV (FEI) at 4 °C and 100 % humidity with Grade 595 Vitrobot™ filter paper (Electron Microscopy Sciences). All grids were plunge-frozen using liquid ethane and stored in liquid nitrogen.

### Data Collection.

Datasets were collected on a Titan Krios (ThermoFisher Scientific), operated at 300 kV, with a K3 direct electron detector and a Bioquantam energy filter (Gatan). Movies were collected at a nominal magnification of 81,000× and a slit width of 20 eV (Table 1). Data collection was carried out using EPU software (Thermo Fisher Scientific).

### Image Processing.

All image processing was carried out in RELION 4.0 (32), using MOTIONCORR implementation in RELION (33) and CTFFIND4 (34) with particles picked using Topaz (35). For RPitc 5nt complexes, the published RPitc complex was used as an initial reference model (EMDB: 4397) (16), whereas the other datasets used the model from the complex containing 1nt shorter RNA length as a reference (e.g., RPitc-5nt as reference for RPitc-6nt dataset). Classes were selected based on definition of key σ^54^ subdomains using a combination of focused 3D classification, density subtraction, and recentering. A range of masks were tested, with the most accurate angular assignments coming from masks that cover CBD and RII, in order to separate σ^54^-bound classes within the dataset, with tighter masks around the DNA–RNA hybrid used in later stages to identify RNA bound complexes.

### Model Building and Refinement.

All structural models were built using COOT (36) and refined using real-space refine in PHENIX (37, 38). All figures were prepared using UCSF ChimeraX (39).

## Supplementary Material

Appendix 01 (PDF)

## Data Availability

The cryoEM maps and coordinates of the initial transcribing complexes described in this work are deposited and available at wwPDB with the following access codes: complex with 5 nt mRNA in a pre-translocated conformation (PDB ID 8RE4 (40), EMD-19079 (41)); complex with 5 nt mRNA in a post-translocated conformation (PDB ID 8REA (42), EMD-19080 (43)); complex with 6 nt mRNA (PDB ID 8REB (44), EMD-19081 (45)); complex with 7 nt mRNA (PDB ID 8REC (46), EMD-19082 (47)); complex with 8 nt mRNA (PDB ID 8RED (48), EMD-19083 (49)); complex with 9 nt mRNA (PDB ID 8REE (50), EMD-19084 (51)). All other study data are included in the article and/or *SI Appendix*.
