# Supplementary material for: Structural basis of σ54 displacement and promoter escape in bacterial transcription
Source: Proc Natl Acad Sci U S A. 2024 Jan 3;121(2):e2309670120. doi: 10.1073/pnas.2309670120 (PMC10786286; doi:10.1073/pnas.2309670120)
Supplement: Supplementary file 1 — Appendix 01 (PDF) [file pnas.2309670120.sapp.pdf]

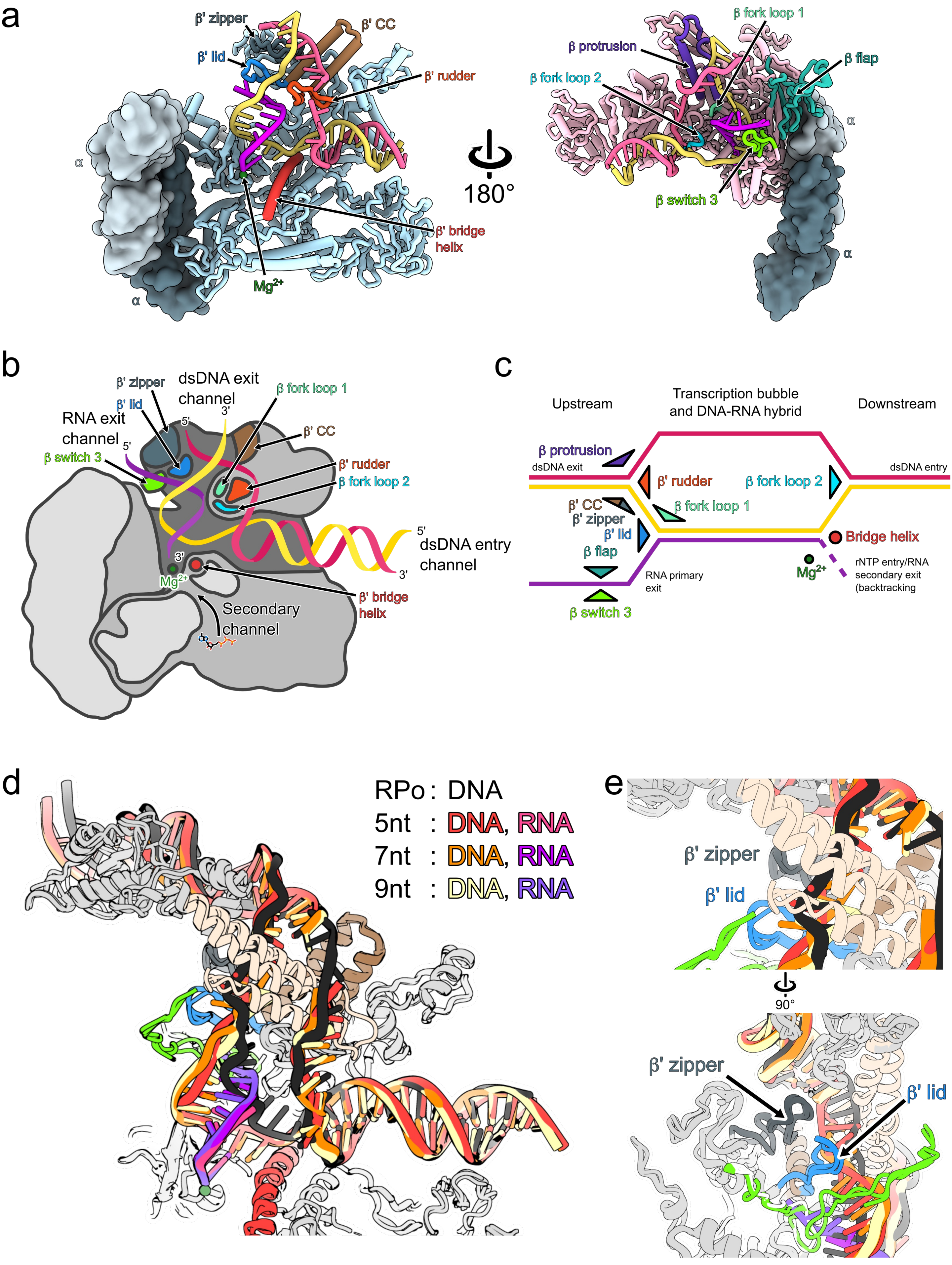

Supplementary Fig. 1 Key conserved features in RNAP. a-c, elongation structure showing regions responsible for maintaining the transcription bubble. Adapted from PDB 6XDR. d, maintenance of the transcription bubble in the initial transcribing complex.

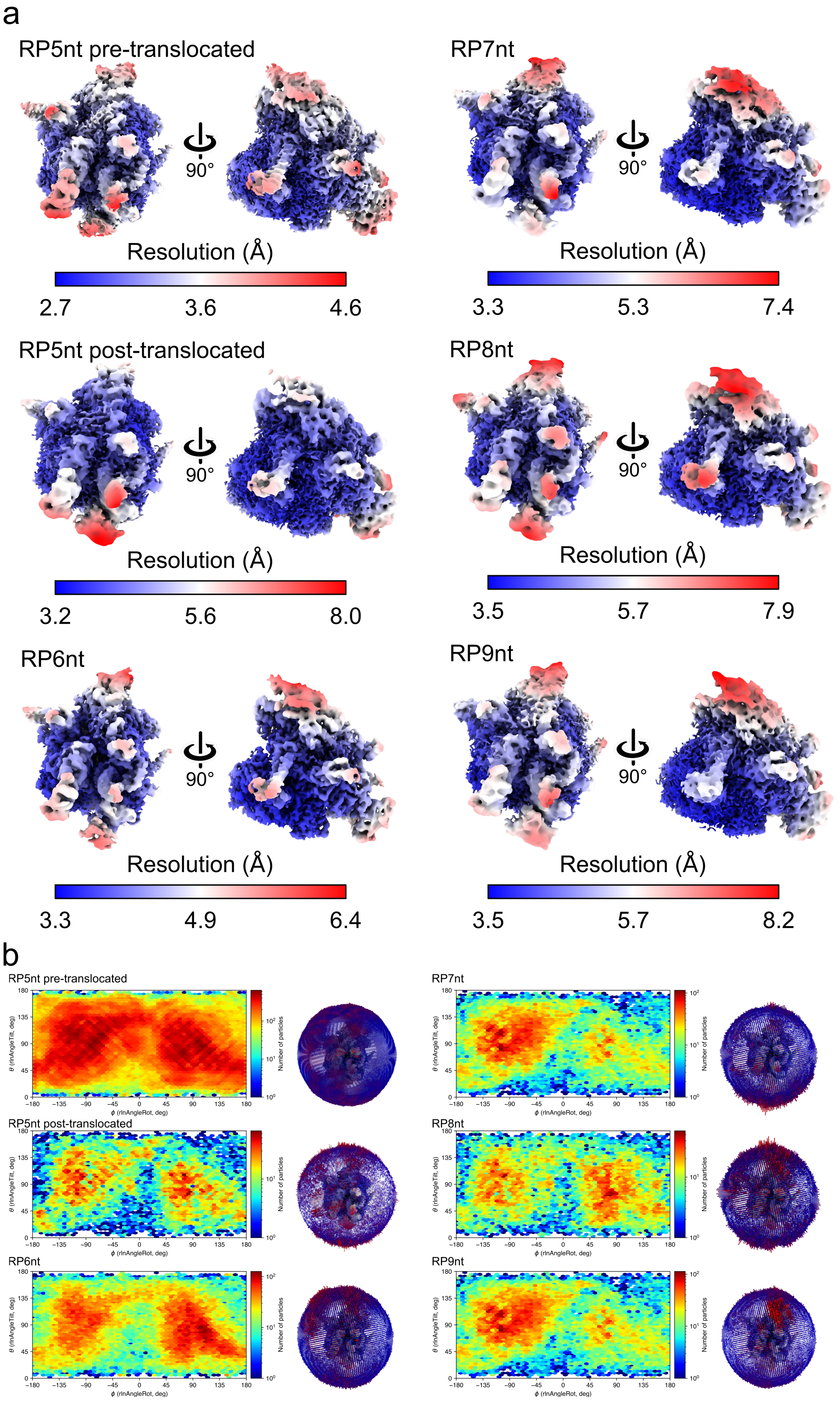

Supplementary Fig. 2. Resolution maps, angular distribution of contributing particles: a) Local resolution maps and b) Angular distribution maps of the final reconstructions.

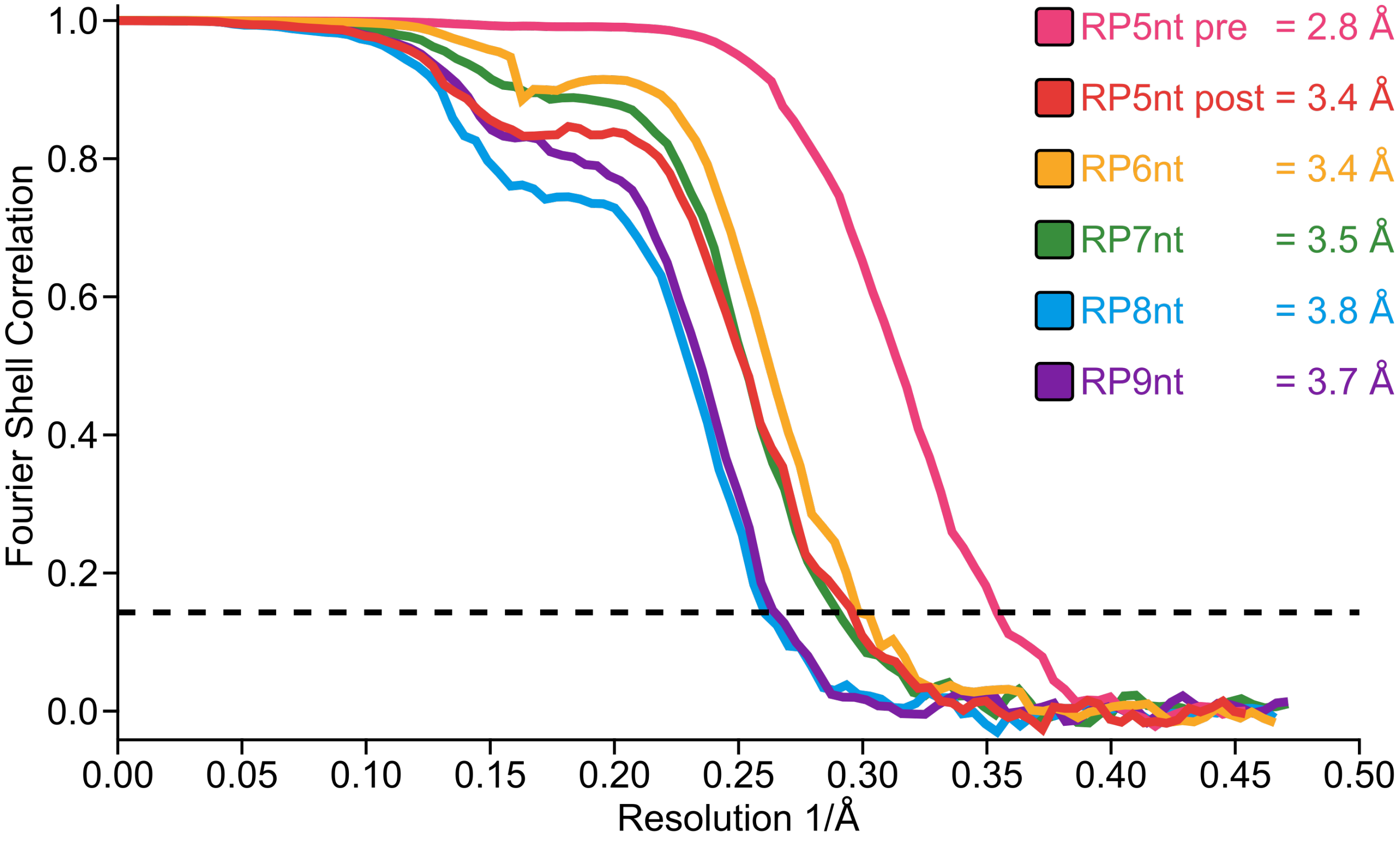

Supplementary Fig. 3: Fourier shell correlation curves (based on corrected maps).
Resolution indicated are based on 0.143 criterium

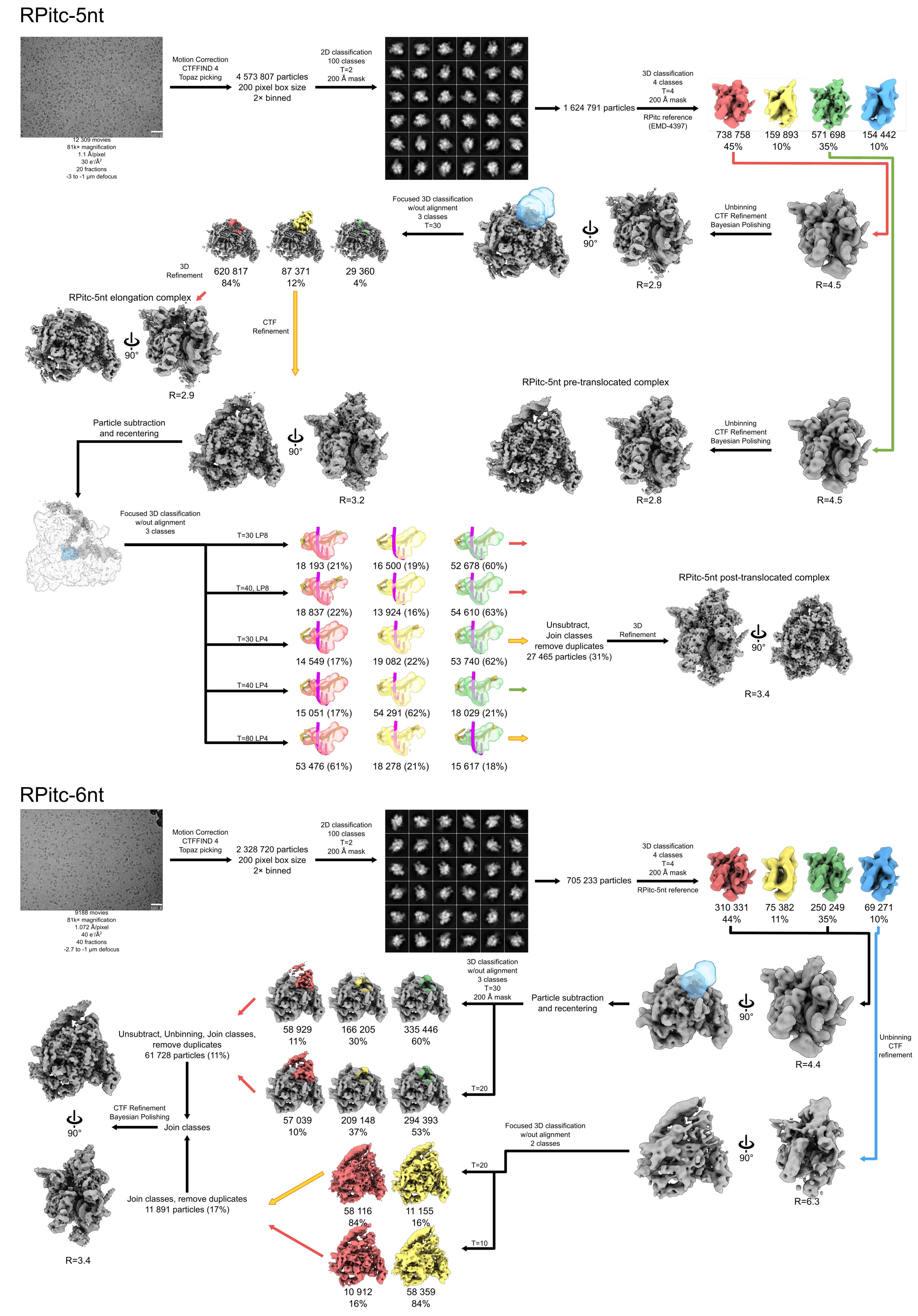

Supplementary Fig. 4: Data processing pipeline for the RPitc 5nt dataset. Colour of arrows indicates the classes chosen.

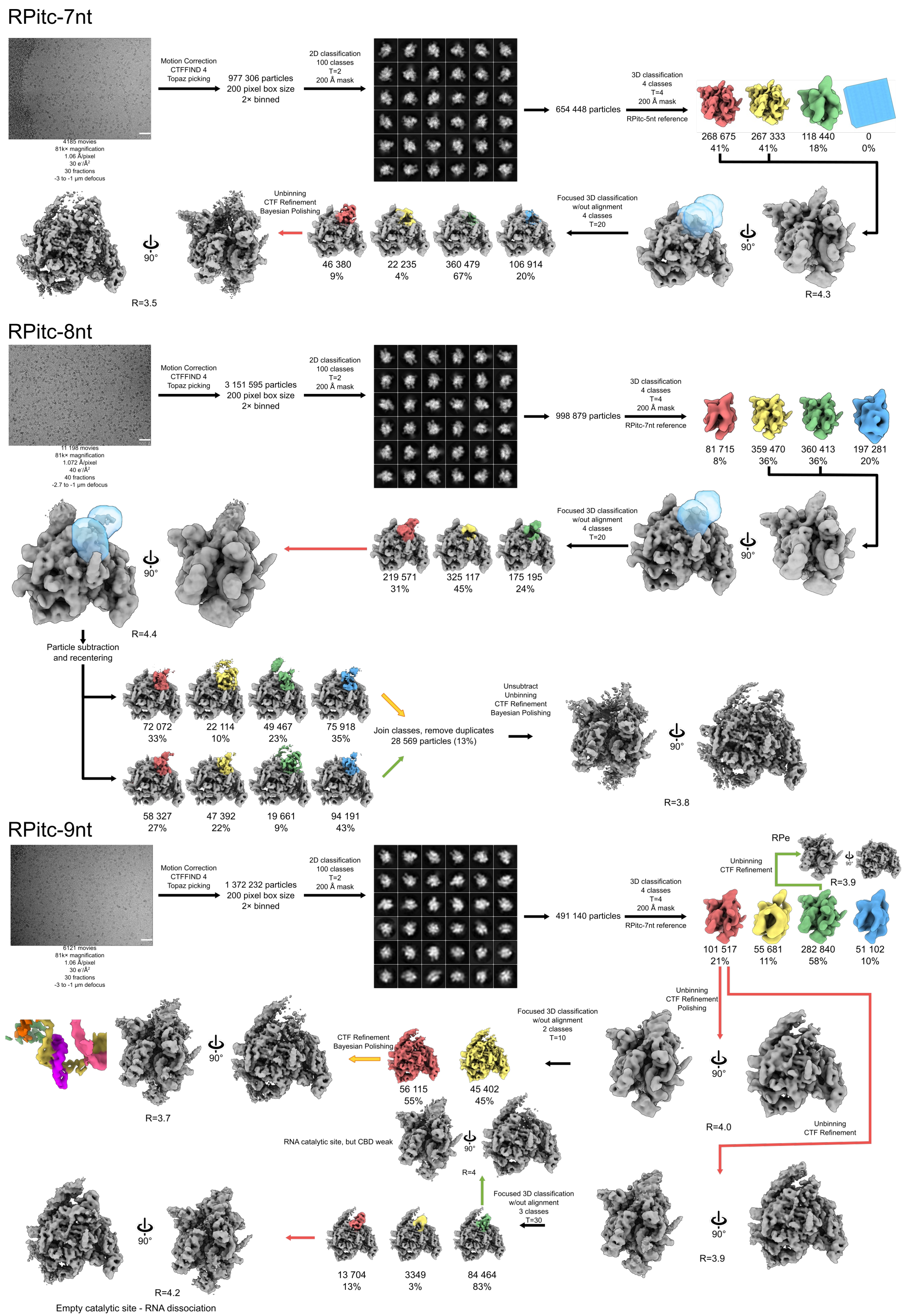

Supplementary Fig. 5: Data processing flowcharts for 7-9nt RNA. Colour of arrows indicates the classes chosen.

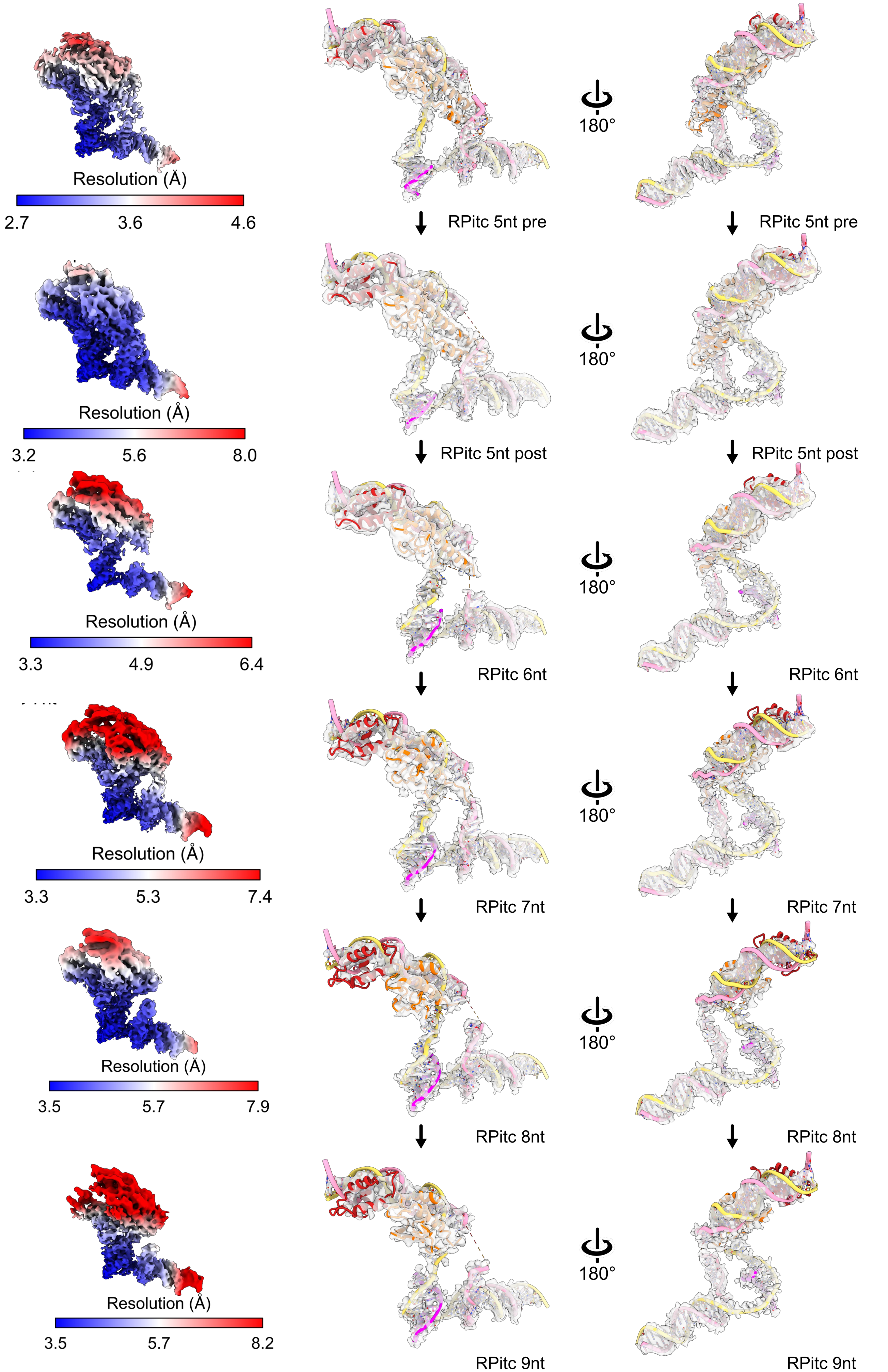

Supplementary Fig. 6: Local resolution and electron density map for DNA, RNA and σ^{54}.

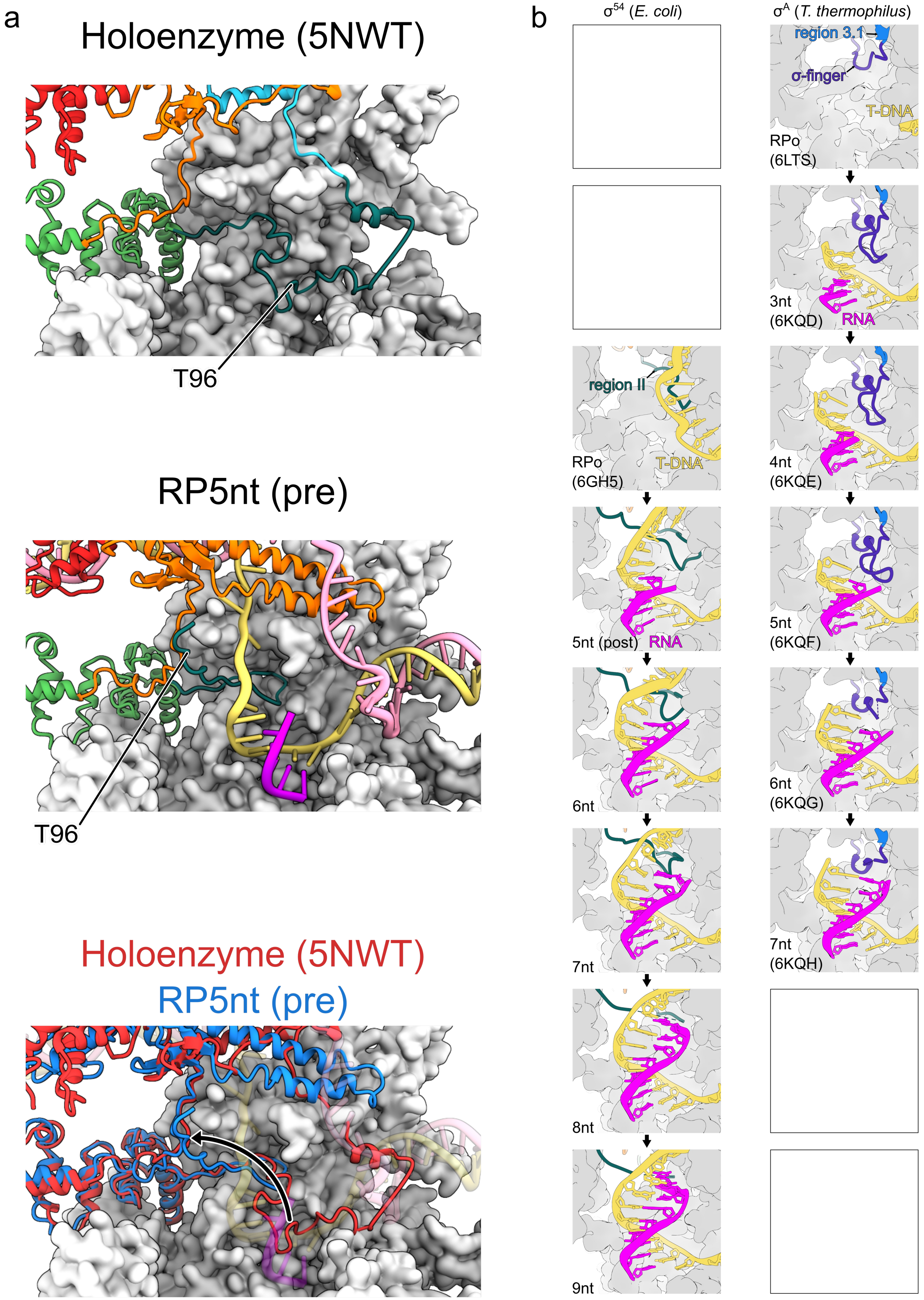

Supplementary Fig. 7: RII-finger conformations. (b) Comparison of RII in holoenzyme (5NWT) and RP5nt (pre) structure showing the relocation of RII upon binding the DNA-RNA hybrid. (b) Comparison of σ^{54} and σ^{70} (σ^{A}) initially transcribing complexes demonstrates that both utilise a similar mechanism of displacement of subdomains blocking the RNA exit channel, in which the 5' end of the growing RNA displaces σ factor, causing it to fold backwards..
